# Epithelial-Mesenchymal Transition-Related MicroRNAs and Their Target Genes in Colorectal Cancerogenesis

**DOI:** 10.3390/jcm8101603

**Published:** 2019-10-03

**Authors:** Branislava Ranković, Nina Zidar, Margareta Žlajpah, Emanuela Boštjančič

**Affiliations:** Faculty of Medicine, Institute of Pathology, University of Ljubljana, Korytkova 2, 1000 Ljubljana, Slovenia

**Keywords:** colorectal adenoma, colorectal carcinoma, metastases, intra-tumour heterogeneity, epithelial-mesenchymal transition, *miR-200* family, target genes

## Abstract

MicroRNAs of the *miR-200* family have been shown experimentally to regulate epithelial-mesenchymal transition (EMT). Although EMT is the postulated mechanism of development and progression of colorectal cancer (CRC), there are still limited and controversial data on expression of *miR-200* family and their target genes during CRC cancerogenesis. Our study included formalin-fixed paraffin-embedded biopsy samples of 40 patients (10 adenomas and 30 cases of CRC with corresponding normal mucosa). Expression of *miR-141*, *miR-200a/b/c* and *miR-429* and their target genes (*CDKN1B*, *ONECUT2*, *PTPN13*, *RND3*, *SOX2*, *TGFB2* and *ZEB2*) was analysed using quantitative real-time PCR. Expression of E-cadherin was analysed using immunohistochemistry. All miRNAs were down-regulated and their target genes showed the opposite expression in CRC compared to adenoma. Down-regulation of the *miR-200* family at the invasive front in comparison to the central part of tumour was observed as well as a correlation of expression of *miR-200b*, *CDKN1B*, *ONECUT2* and *ZEB2* expression to nodal metastases. Expression of the *miR-200* family and *SOX2* also correlated with E-cadherin staining. These results suggest that the *miR-200* family and their target genes contribute to progression of adenoma to CRC, invasive properties and development of metastases. Our results strongly support the postulated hypotheses of partial EMT and intra-tumour heterogeneity during CRC cancerogenesis.

## 1. Introduction

Colorectal cancer (CRC) is one of the most common cancers worldwide. Five-year survival for patients with early CRC is approximately 90%, while for patients with advanced CRC, survival drops to 8%–12%. The prognosis can improve significantly with the introduction of population screening programs; however, 40%–50% of CRC patients still develop metastases [1,2]. Cancerogenesis of CRC is divided into well-established discrete stages, from normal mucosa to invasive carcinoma. The majority of CRC develops from precursor lesions—adenomas. The molecular pathways that are responsible for transformation of normal mucosa to adenoma and CRC are well understood and include stepwise accumulation of mutations (microsatellite instability or MSI pathway; chromosome instability or CIN pathway), epigenetic changes (CpG island methylator phenotype, CIMP) and changes in gene expression [3,4]. The majority of events occur before the formation of adenoma. Despite extensive research, the role of epithelial-mesenchymal transition (EMT) remains one of the controversial aspects of CRC development from normal mucosa to adenoma and carcinoma. EMT is believed to be one of the key processes in development of metastases in CRC, being responsible for the increased motility of cancer cells at the invasive front [5,6,7].

EMT is one of the crucial processes in embryonal development, being essential for morphogenesis and organ development [5]. In adult life, it contributes to physiological and pathological processes, such as wound healing, tissue regeneration, organ fibrosis and development and progression of malignant tumours. During EMT, epithelial cells undergo extensive changes that lead to separation of cells, re-organization of the extracellular matrix and an increase in cell motility, and invasion [6,7,8,9].

EMT is difficult to observe at a molecular level due to the reversible nature of changes, present only in a minority of cells [6,7,9]. Several markers of EMT have been described since its postulated contribution to cancer development [5,6,7,8,9,10]. Besides up-regulation of transcriptional factors of EMT, several miRNAs have been found to be involved in EMT regulation, the most frequent finding being down-regulation of the *miR-200* family (*miR-200a*, *miR-200b*, *miR-200c*, *miR-141*, *miR-429*), which is an important feature of EMT [2,6]. Transcription factors of EMT and their regulators are thought to support all cancer stages: from tumour initiation, establishment of precancerous lesion, accumulation of genetic alterations and escape from tumour surveillance and development of metastases [11].

Despite numerous publications suggesting that EMT might be responsible for metastases development in CRC [2,10,12], there is limited data about the involvement of EMT, including the *miR-200* family and their target genes [13], at early stages of CRC cancerogenesis. There is also limited data on differential expression of the *miR-200* family in different parts of the tumour, i.e., at the invasive front of CRC in comparison to the central part of the tumour, suggesting intra-tumour heterogeneity (ITH). ITH has emerged as an important phenomenon in cancer and it is related to different morphologic and phenotypic profiles of tumour cell in various parts of the tumour, including cellular morphology, gene expression and (epi)genetic/genomic aberrations, as well as metastatic potential. It is believed to contribute to cancer progression, resistance to therapy and recurrences [14]. ITH of the *miR-200* family might contribute to a lower expression of epithelial markers and gain of mesenchymal markers at the invasive front [13].

We therefore hypothesized that EMT in CRC might be responsible for malignant transformation of adenoma to carcinoma, development of metastases to the regional lymph nodes and ITH. Our aim was to investigate expression of the *miR-200* family and their target genes in CRC cancerogenesis from normal mucosa to adenoma and carcinoma without and those with nodal metastases. To the best of our knowledge, there has been no research systematically exploring the involvement of the *miR-200* family and their target genes in all stages of CRC development.

## 2. Experimental Section

### 2.1. Tissue Samples

Tissue samples from 40 patients with adenoma and CRC were included in the study. For routine histopathologic examination, tissue samples were fixed in 10% buffered formalin and embedded in paraffin (FFPE). CRC specimens were evaluated according to standard procedures and after histopathologic examination, pTNM (pathologic Tumour Node Metastasis) classification was assessed on the basis of the depth of invasion and extent of the primary tumour, the number of lymph nodes with metastases, and the presence of distant metastases [15]. Samples were collected retrospectively from the archives of the Institute of Pathology, Faculty of Medicine, University of Ljubljana. For all patients, tumour samples and samples of normal mucosa (if available) were included. Patients treated either by radiotherapy, chemotherapy or biologic drugs prior to surgery were excluded from the study. On the basis of clinical and histopathological features, samples were divided into three groups: patients with adenoma (*n* = 10), patients with carcinoma without nodal metastases (CRC N0, *n* = 13), patients with carcinoma with nodal metastases (CRC N+, *n* = 17).

In all cases, EMT was evaluated based on the expression of E-cadherin, *miR-200* family and miRNAs target genes. For the purpose of the study, adenomas were compared to carcinoma and normal colon mucosa. Carcinoma with regional lymph node metastases were compared to those without lymph node metastases. ITH was analysed comparing the invasive front of the tumour and the centre of the tumour in both CRC N0 and CRC N+ groups.

The investigation was carried out following the rules of the Declaration of Helsinki. The study was approved by the National Medical Ethics Committee (Republic of Slovenia, Ministry of Health). 

### 2.2. Immunohistochemistry

FFPE tissue samples were cut at 4 µm for immunohistochemistry. All reagents were from Ventana Medical Systems Inc. (Tuscon, AZ, USA) except where otherwise indicated. Commercially available antibodies against E-cadherin (Dako Agilent, Santa Clara, CA, USA, M3612, clone NC4-38, dilution 1:10) were used. Deparaffinization, antigen retrieval and staining were performed in an automatic immunostainer (Benchmark XT, Ventana, Tuscon, AZ, USA) using horseradish peroxidase (iVIEW DAB Detection Kit, Roche, Basel, Switzerland) for colour development. The sections were then counterstained with haematoxylin.

### 2.3. RNA Isolation from Formalin-Fixed Paraffin Embedded (FFPE) Tissue Samples

#### 2.3.1. RNA Isolation from FFPE Tissue Slides

Tissue samples were cut at 10 μm from FFPE tissue blocks and four sections were used for the isolation procedure. Total RNA isolation was performed using an AllPrep DNA/RNA FFPE kit (Qiagen, Hilden, Germany) according to the manufacturer’s protocol. The concentration and quality of the isolates were assessed with a spectrophotometer ND-1000 (Nanodrop, Thermo Fisher Scientific, Waltham, MA, USA) at wavelengths 260, 280 and 230 nm. 

#### 2.3.2. RNA Isolation from FFPE Tissue Cores (Punched) Samples

For analysis of ITH, tumour samples were punched from FFPE tissue blocks (from invasive front and central part of tumour) using a 600 μm needle. For the isolation procedure, 3 punches were used from each tumour region. Total RNA isolation was performed using a MagMax FFPE DNA/RNA Ultra kit (Applied Biosystems, Foster City, CA, USA) according to the manufacturer’s protocol with one modification. Protease digestion was performed overnight at 56 °C with shanking for 15 s at 300 rpm every 4 min. The concentration and quality of the isolates were assessed with a spectrophotometer ND-1000 (Nanodrop, Thermo Fisher Scientific, Waltham, MA, USA) at the wavelengths 260, 280 and 230 nm. 

#### 2.3.3. RNA Quality Assessment

Reverse transcription (RT) followed by amplification of the *GAPDH*, a housekeeping gene (100 base pairs), using quantitative real-time PCR (qPCR) and Sybr Green technology, was used as quality control. All of the samples included in the study had passed amplification of *GAPDH* (initially quality control) and those that did not amplify were not included in the study (we isolated at least twice as many samples as are included within this manuscript). Second, for selected genes, we chose TaqMan primers and probes that amplify and detect PCR products less than 100 bp long (Table 1). 

### 2.4. Analysis of Expression of Family miR-200 and miR-205

miRNAs family *miR-200* was analysed using qPCR based on the TaqMan methodology (Thermo Fisher Scientific, Waltham, MA, USA). A pre-designed mixture of probes and primers specific for target miRNAs expression was used. Prior to qPCR, three pools of RNA samples were created, obtained from normal mucosa, adenomas and advanced CRC. After RT, the cDNA was diluted in five steps, ranging from 4-point dilution to 1024-point dilution, and the probes were tested for qPCR efficiency. All the qPCR efficiency reactions were performed on a RotorGene Q (Qiagen, Hilden, Germany) in triplicate.

#### 2.4.1. Reverse Transcription (RT)

Looped primers for specific reverse transcription (RT) of miRNAs and a MicroRNA TaqMan RT kit (Applied Biosystems, Foster City, CA, USA) were utilized following the manufacturer’s protocol. *RNU6B* and *miR-1247b* were used as reference genes (RGs). MicroRNAs, *miR-141*, *miR-200a*, *miR-200b*, *miR-200c* and *miR-429* were tested relative to the geometric mean of expression of *RNU6B* and *miR-1247b* (Table 1). Briefly, a 10 μL RT reaction master mix was performed with 10 ng of total RNA sample, 1.0 μL of MultiScribe Reverse Transcriptase (50 U/μL), 1.0 μL of Reverse Transcription Buffer (10×), 0.1 μL of dNTP (100 mM), 0.19 μL RNAase inhibitor (20 U/μL), and 2.0 μL of RT primer (5×). The reaction conditions were: 16 °C for 30 min, 42 °C for 30 min, 85 °C for 5 min. 

#### 2.4.2. Quantitative Real-Time PCR (qPCR)

qPCR for miRNAs was carried out in a 10 μL PCR master mix containing 5.0 μL TaqMan 2× FastStart Essential DNA Probe Master (Roche, Basel, Switzerland), 0.5 μL TaqMan assay and 4.5 μL RT products diluted 100-fold. The qPCR reactions were performed on a RotorGene Q (Qiagen, Hilden, Germany) in duplicate, as follows: initial denaturation at 95 °C for 10 min, 40 cycles for 15 s at 95 °C (denaturation) and for 60 s at 60 °C (primers annealing and elongation). The signal was collected at the endpoint of every cycle. 

### 2.5. Analysis of Expression of miR-200 Family Target Genes

mRNA expression of protein-coding genes was analysed using qPCR based on the TaqMan methodology (Thermo Fisher Scientific, Waltham, MA, USA). A pre-designed mixture of probes and primers specific for target mRNAs expression was used. Prior to qPCR, four pools of RNA samples were created, obtained from normal mucosa, adenomas, advanced CRC without and CRC with nodal metastases. After RT and PreAmp, the pre-amplified cDNA was diluted in four steps, ranging from 5-point dilution to 625-point dilution, and the probes were tested for qPCR efficiency. All the qPCR efficiency reactions were performed on a RotorGene Q (Qiagen, Hilden, Germany) in triplicate.

#### 2.5.1. Reverse Transcription (RT)

Target mRNAs of the *miR-200* family, *CDKN1B*, *ONECUT2*, *PTPN13*, *RND3*, *SOX2*, *TGFB2*, *WAVE3*, *ZEB1* and *ZEB2* (Table 1), were analysed relatively to the geometric mean of RGs, *IPO8* and *B2M*. mRNAs were reverse transcribed using a OneTaq RT-PCR Kit (New England Biolabs, Ipswich, MA, USA) using random primers according to the manufacturer’s instructions. Reverse transcription reactions were started with 3.0 µL (60 ng) of total RNA and 1.0 µL of Random Primer Mix incubated at 70 °C for 5 min. The 10 μL RT master mix included 5.0 μL of M-MuLV Reaction Mix, 1.0 μL of M-MuLV reverse transcriptase and 4.0 μL of reaction mix after random priming. The reaction conditions were: 25 °C for 5 min, t 42 °C for 60 min and 80 °C for 4 min. 

#### 2.5.2. Pre-Amplification and Quantitative Real-Time PCR (qPCR)

Following RT, pre-amplification was performed using a TaqMan PreAmp Master Mix (Applied Biosystems, Foster City, CA, USA) in 10 µL according to the manufacturer’s protocol. The resulting PreAmp reaction was diluted 5-fold and 4.5 μL was used in a 10 μL reaction volume with a 5.0 μL of 2x FastStart Essential DNA Probe Master Mix (Roche, Basel, Switzerland) and 0.5 μL of TaqMan probe. Thermal conditions were applied as follows: 50 °C for 2 min, initial denaturation at 95 °C for 10 min and 40 cycles of denaturation at 95 °C for 15 s and annealing at 60 °C for 1 min. All qPCR analyses were performed on a Rotor Gene Q (Qiagen, Hilden, Germany) in duplicate. The signal was collected at the endpoint of each cycle. 

### 2.6. Statistical Analysis of Experimental Data

The results were presented as relative gene expression. All Cqs were corrected for PCR efficiencies and the expression of the gene of interest (GOI, Cq_GOI_) was calculated relative to a geometric mean of RGs (Cq_RG_), named ΔCq. In CRC samples, mRNAs and miRNAs expression differences were compared between tumours and adjacent normal tissue using ΔCq and the Willcoxon Rank test. For comparison of relative quantification of mRNA and miRNA between independent groups of samples (i.e., adenomas and normal mucosa), ΔCq and the Mann-Whitney test were used. The same test was used for comparison of tumours with nodal metastases to those without nodal metastases, except that ΔΔCq was used. For all correlations/associations, Spearman rank-order correlation was used. Statistical analysis of data was performed using SPSS version 24 (SPSS Inc., Chicago, IL, USA). Differences were considered to be significant at *p* < 0.05. 

## 3. Results

### 3.1. Patients and Tissue Samples

The group of adenomas included 10 patients, the group of CRC without lymph node metastases (CRC N0) included 13 patients and the group of CRC with lymph node metastases (CRC N+) included 17 patients. As a control group, microscopically normal colon mucosa from surgical margins from 30 patients with CRC N0 and CRC N+ was used. Sex, age and location for each group are presented in Table 2. Only cases with a clear-cut biopsy diagnosis were included. In the group of adenomas, there were four tubular adenomas with high grade dysplasia, four tubulovillous adenomas with high grade dysplasia and two tubulovillous adenomas with low grade dysplasia. 

All tissue samples were fixed for 24 h in 10% buffered formalin prior to paraffin embedding. After fixation and embedding, tissues were cut into 3–4 µm slides and stained with haematoxylin and eosin for routine histopathological examination. For the purposes of our study, representative paraffin blocks were collected from the archives of the Institute of Pathology, Faculty of Medicine, University of Ljubljana.

### 3.2. Immunohistochemistry and Expression of E-Cadherin

**Figure 1:**
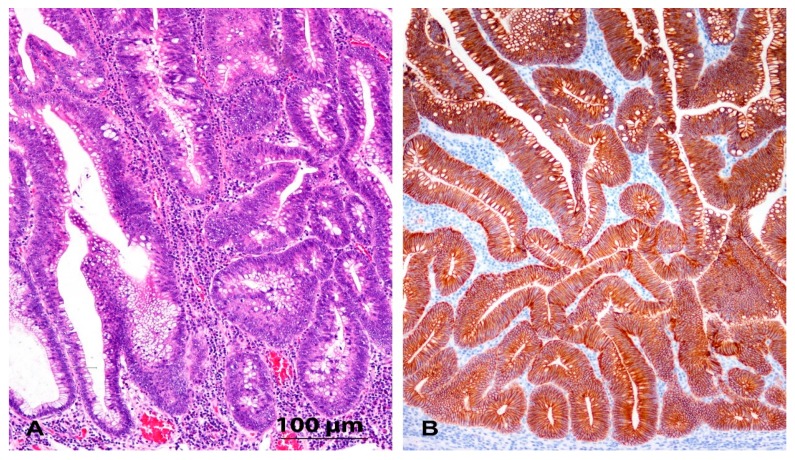
(**A**) Tubular adenoma. HE, orig. magnification 100×; (**B**) Immunohistochemistry for E-cadherin in adenoma (*n* = 10): diffuse, strong membranous reaction. Orig. magnification 100×.

**Figure 2:**
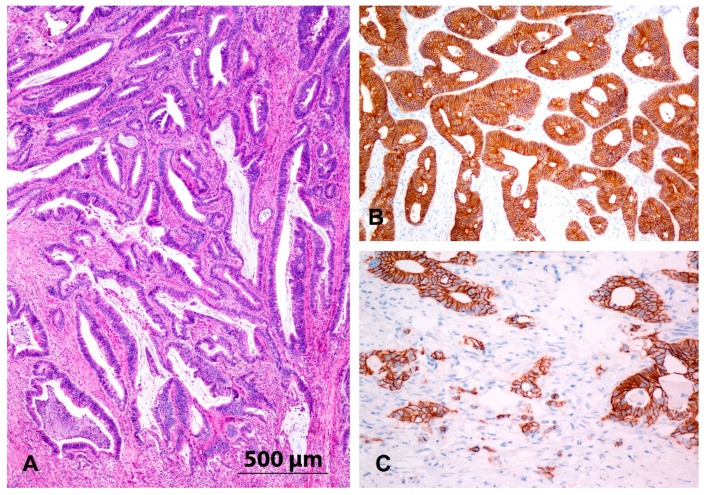
(**A**) Moderately differentiated adenocarcinoma. HE, orig. magnification 40×; (**B**,**C**) Immunohistochemistry for E-cadherin in adenocarcinoma (*n* = 30): strong membranous reaction in central part of the tumour (**B**) and focally reduced staining at the invasive tumour front (**C**). Orig. magnification 100×.

E-cadherin staining was preserved in the normal colon mucosa and mostly in adenomas and carcinomas. However, in a proportion of samples of adenoma and carcinoma, we observed focal loss or weak staining of E-cadherin at the periphery of the lesion or the invasive front. All four cases of adenoma with decreased E-cadherin staining showed high-grade dysplasia. The staining of E-cadherin was also decreased in seven of 13 cases with CRC N0 and in nine of 17 cases with CRC N+. The results are summarized in Figure 1 and Figure 2 and Table 2.

Microscopic analysis of the CRCs and adenomas showed that all cases retained an epithelioid morphology, even those with a decreased expression of E-cadherin. No spindle cell morphology was found in any case of adenoma or CRC, either in the central parts or at the invasive front (Figure 1 and Figure 2).

### 3.3. Undetectable Expression of Markers of EMT

The expression level of *miR-205*, *ZEB1* (target of *miR-200* family) and *WAVE3* (target of *miR-200b*) was beyond the detection limit when analysing the amplification efficiency on pooled samples. These EMT markers were therefore omitted from further analysis. Expression of *miR-205*, *ZEB1* and *WAVE3* was beyond the limit of detection in all groups, i.e., normal mucosa, adenoma, CRC N0 and CRC N+.

### 3.4. Expression of the miR-200 Family and Its Target Genes in Adenoma Compared to Normal Colon Mucosa

The geometric mean of expression of RGs for miRNA was comparable between adenoma and normal mucosa of patients with CRC N0, but different from the geometric mean of expression in normal mucosa of patients with CRC N+. All the comparisons of adenoma to normal mucosa were therefore performed only with normal mucosa of CRC N0. We also observed a statistical difference for four out of five investigated miRNAs when comparing normal mucosa of CRC N0 with normal mucosa of CRC N+. This observation further supported our selection of normal mucosa samples for comparison of miRNAs and mRNAs expression to that in adenoma. 

**Figure 3:**
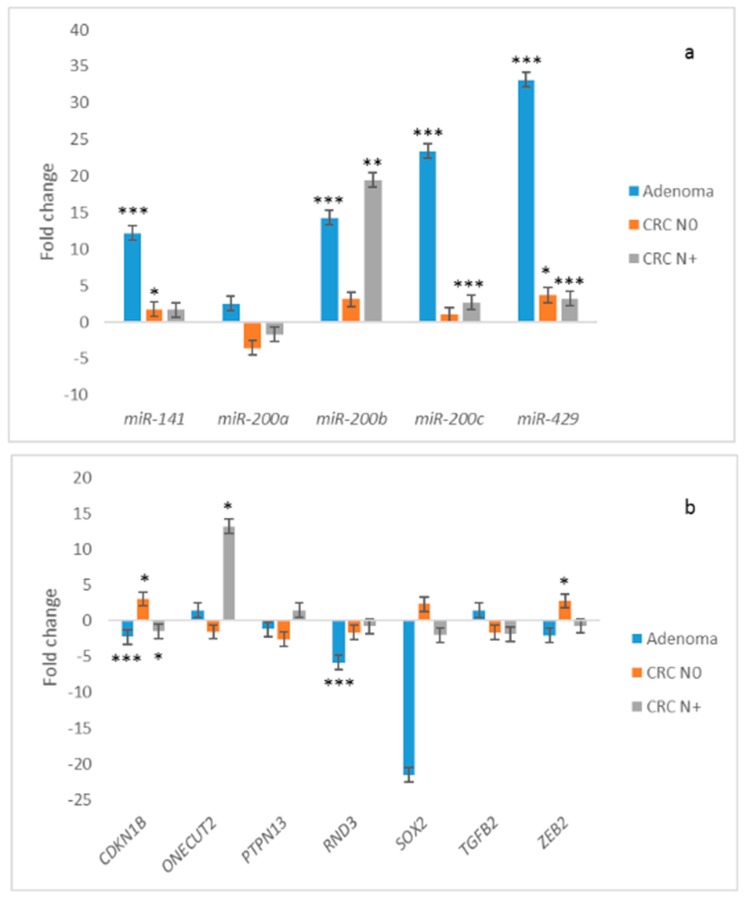
Expression of *miR-200* family and their target genes in adenoma (*n* = 10), CRC N0 (*n* = 13) and CRC N+ (*n* = 17) in comparison with normal mucosa (*n* = 13, *n* = 13 and *n* = 17, respectively): (**a**) Expression of *miR-200* family; (**b**) expression of target genes of *miR-200* family. Legend: CRC, colorectal carcinoma; N0, without nodal metastases; N+, with nodal metastases; * *p* ≤ 0.05; ** *p* ≤ 0.01; *** *p* ≤ 0.001.

The expression of all miRNAs was up-regulated in adenoma compared to normal mucosa of CRC N0. Up-regulation was statistically significant in the case of *miR-141* (~12.2-fold, *p* = 0.001), *miR-200b* (~14.3-fold, *p* < 0.001), *miR-200c* (~23.4-fold, *p* < 0.001) and *miR-429* (~33.2-fold, *p* < 0.001). Results are summarized in Figure 3a.

All investigated and expressed target genes, *CDKN1B*, *PTPN13*, *RND3*, *SOX2* and *ZEB2*, were down-regulated in adenoma compared to normal mucosa of CRC N0, except *ONECUT2* and *TGFB2*, which were up-regulated. Moreover, *PTPN13*, *SOX2* and *ZEB2* were expressed only in two out of 10 samples of adenoma, so a calculation of statistical significance would not be appropriate. To summarize, down-regulation reached statistical significance only in the case of *CDKN1B* (~2.3-fold, *p* = 0.015) and *RND3* (~5.9-fold, *p* < 0.001), both targets of *miR-200b*. Results are summarized in Figure 3b.

We also observed a statistically significant strong negative correlation between the expression of *CDKN1B* and *miR-200a* (r_s_ = −0.648, *p* = 0.043) in adenomas.

### 3.5. Expression of the miR-200 Family and Its Target Genes in Carcinoma without Nodal Metastasis Compared to Normal Mucosa

Expression of all miRNAs was up-regulated in CRC N0 compared to corresponding normal mucosa, except *miR-200a*, which was down-regulated. Up-regulation was statistically significant in the case of *miR-141* (~1.7-fold, *p* = 0.019) and *miR-429* (~3.7-fold, *p* = 0.041). Results are summarized in Figure 3a.

In contrast to adenoma, investigated target genes *CDKN1B*, *PTPN13*, *RND3*, *SOX2* and *ZEB2* were up-regulated in CRC N0, except *ONECUT2* and *TGFB2*, which were down-regulated. Statistically significant up-regulation was observed for *CDKN1B* (~3.0-fold, *p* = 0.015) and for *ZEB2* (~2.7-fold, *p* = 0.011). Results are summarized in Figure 3b.

**Figure 4:**
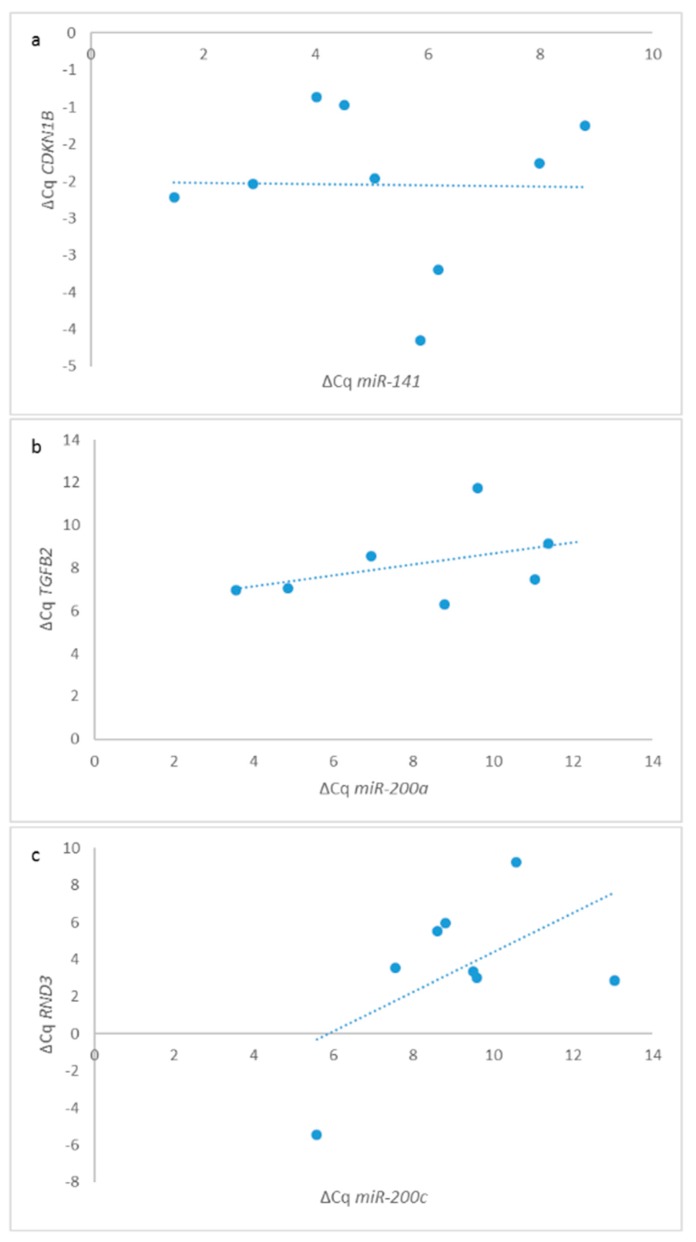
Correlations of expression of *miR-200* family and their target genes in CRC N0 (*n* = 10): (**a**) correlation between *miR-141* and *CDKN1B*; (**b**) correlation between *miR-200a* and *TGFB2*; (**c**) correlation between *miR-200c* and *RND3*.

The Spearman coefficient of correlation showed that in CRC N0, expression of *miR-200a* was in correlation with the expression of *TGFB2* (r_s_ = 0.900, *p* = 0.037), *CDKN1B* was in correlation with *miR-141* (r_s_ = 0.683, *p* = 0.042) and *RND3* to *miR-200c* (r_s_ = 0.867, *p* = 0.002). All correlations were positive and strong or very strong. Results are presented in Figure 4.

### 3.6. Expression of the miR-200 Family and Its Target Genes in Carcinoma with Nodal Metastasis Compared to Normal Mucosa

The expression of all miRNAs, except *miR-200a*, was up-regulated in CRC N+ compared to corresponding normal mucosa; up-regulation was statistically significant in the case of *miR-200b* (~19.4-fold, *p* < 0.001), *miR-200c* (~2.7-fold, *p* = 0.003) and *miR-429* (~3.2-fold, *p* = 0.006). Results are summarized in Figure 3a.

Expression of miRNAs’ targets was more heterogeneous in CRC N+ than in adenoma and CRC N0. *PTPN13*, *ONECUT*, *SOX2* and *RND3* were up-regulated, and *TGFB2*, *ZEB2* and *CDKN1B* showed down-regulation in CRC N+ compared to its corresponding normal mucosa. Statistical significance was reached only in the case of *CDKN1B* (~1.5-fold, *p* = 0.017) and *ONECUT* (~13.1-fold, *p* = 0.018). Results are summarized in Figure 3b.

The Spearman coefficient of correlation showed that *miR-200a*, *miR-200c* and *miR-141* were expressed in negative correlation with *TGFB2* in CRC N+ (r_s_ = −0.841, *p* = 0.005; r_s_ = −0.765, *p* = 0.016; r_s_ = −0.668, *p* = 0.049; respectively).

### 3.7. Expression of miRNAs and Their Target Genes in Adenomas Compared to Carcinomas

**Figure 5:**
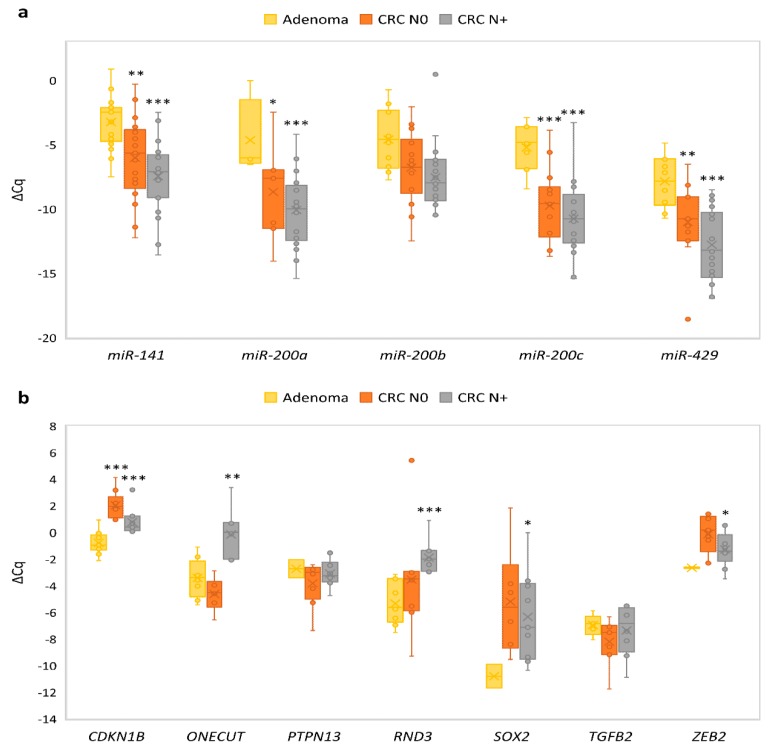
Expression of the *miR-200* family and their target genes in adenoma (*n* = 10) in comparison with CRC N0 (*n* = 13) and CRC N+ (*n* = 17): (**a**) expression of the *miR-200* family; (**b**) expression of *miR-200* family target genes. Legend: CRC, colorectal carcinoma; ΔCq, delta quantitation cycle; N0, without nodal metastases; N+, with nodal metastases; * *p* ≤ 0.05; ** *p* ≤ 0.01; *** *p* ≤ 0.001.

We observed a statistically significant difference in the expression patterns of *miR-200* family and their target genes when adenomas were compared to CRC N0 or CRC N+. Four miRNAs were differentially expressed between adenomas and either CRC N0 or CRC N+ (*miR-200a*, *p* = 0.026 and *p* < 0.001, respectively; *miR-200c*, *p* = 0.001 and *p* < 0.001, respectively; *miR-141*, *p* = 0.007 and *p* < 0.001, respectively; *miR-429*, *p* = 0.011 and *p* < 0.001, respectively); *miR-200b* was differentially expressed only between adenomas and CRC N+ (*p* = 0.003). Results are summarized in Figure 5a.

In contrast to miRNA, the only miRNAs target gene that was differentially expressed between adenomas and both CRC N0 and CRC N+ was *CDKN1B* (*p* < 0.001 and *p* = 0.001, respectively). In adenomas, differential expression in comparison with CRC N0 was also observed for *ZEB2* (*p* = 0.034) and *SOX2* (*p* = 0.046), and in comparison with CRC N+, *ONECUT2* (*p* = 0.006) and *RND3* (*p* < 0.001). Results are summarized in Figure 5b.

### 3.8. Comparison of Expression of miRNAs and Its Target Genes in Carcinoma with Nodal Metastases to Carcinoma without Nodal Metastasis

**Figure 6:**
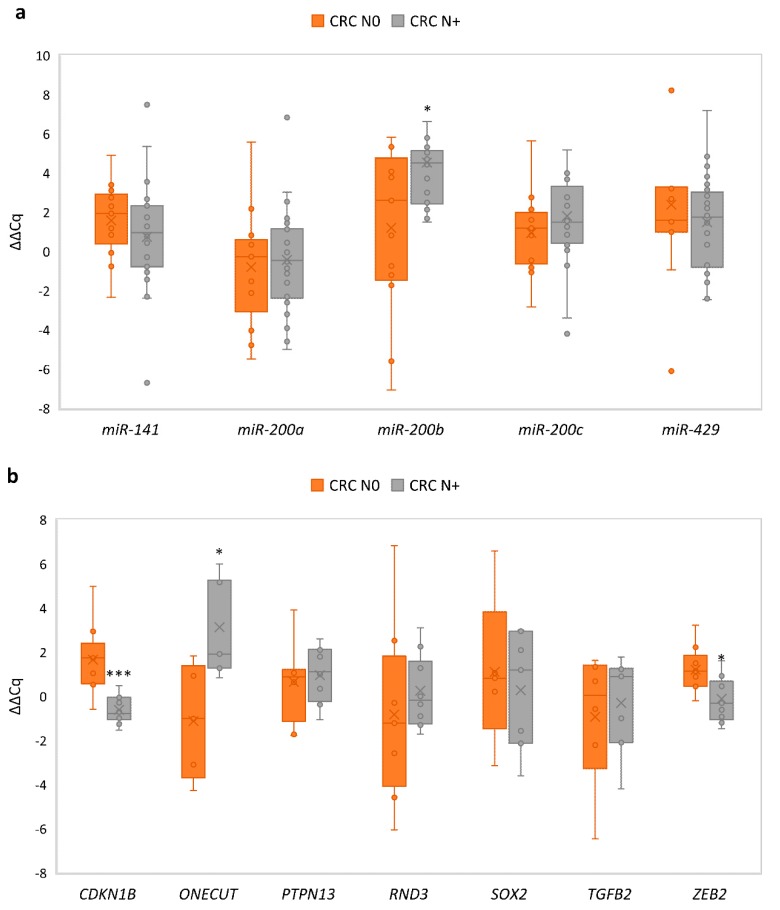
Expression of the *miR-200* family and their target genes in CRC N0 (*n* = 13) in comparison with CRC N+ (*n* = 17): (**a**) expression of the *miR-200* family; (**b**) expression of *miR-200* family target genes. Legend: CRC, colorectal carcinoma; ΔΔCq, delta quantitation cycle; N0, without nodal metastases; N+, with nodal metastases; * *p* ≤ 0.05; *** *p* ≤ 0.001.

Expression in each carcinoma sample was normalized to its corresponding normal mucosa. Groups of CRC N0 and CRC N+ were then compared with each other. It was shown that there was no significant change in expression of investigated miRNAs between CRC N+ compared with CRC N0, except for *miR-200b*. Results are summarized in Figure 6a.

Target genes of the *miR-200* family, *ONECUT*2 showed up-regulation in CRC N+ compared with CRC N0 (*p* = 0.028), whereas *ZEB2* and *CDKN1B* showed down-regulation in CRC N+ compared with CRC N0 (*p* = 0.038 and *p* = 0.001, respectively). Results are summarized in Figure 6b.

### 3.9. Tumour Heterogeneity-Expression of the miR-200 Family in the Central Parts of Carcinoma Compared to the Invasive Front

In a subset of CRC N0 (*n* = 7) and CRC N+ samples (*n* = 8), there was enough material to obtain tissue cores from the central parts and invasive front of the tumour. All miRNAs were mainly down-regulated at the invasive front compared with central part of the tumour.

In the case of CRC N0, four out of seven samples showed down-regulation at the invasive front compared with the central part of tumour, with no statistically significant change in expression. In CRC N+, seven out of eight samples showed down-regulation at the invasive front compared with the central part, with certain miRNA expression being absent, i.e., *miR-200c*. A statistically significant difference in expression between the invasive front and central parts of the tumour was observed for *miR-200b* (*p* = 0.028) and *miR-429* (*p* = 0.028). 

Additionally, the expression of *miR-200b* was significantly differentially expressed when invasive front (normalized to the central parts of the tumour) was compared between CRC N0 and CRC N+ (*p* = 0.046). 

**Figure 7:**
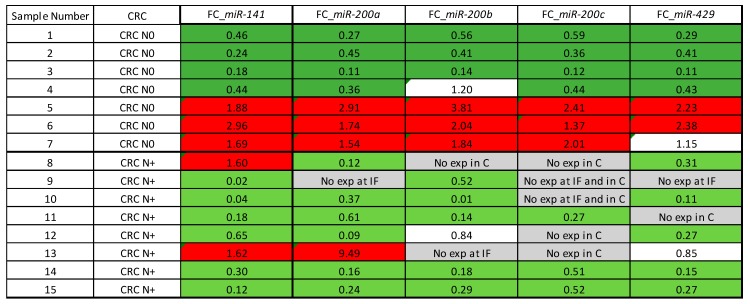
Heat-map of *miR-200* family expression at the invasive front in comparison with central parts of the CRC. Legend: C, central part of the tumour; CRC, colorectal cancer; exp, expression; FC, fold change; IF invasive front; N0, without nodal metastases; N+, with nodal metastases.

Results are presented in Figure 7 as a heat-map of FC between invasive front and central parts for each sample.

### 3.10. Expression of E-Cadherin and Correlation of Its Expression to the Expression of miRNAs and mRNAs

**Figure 8:**
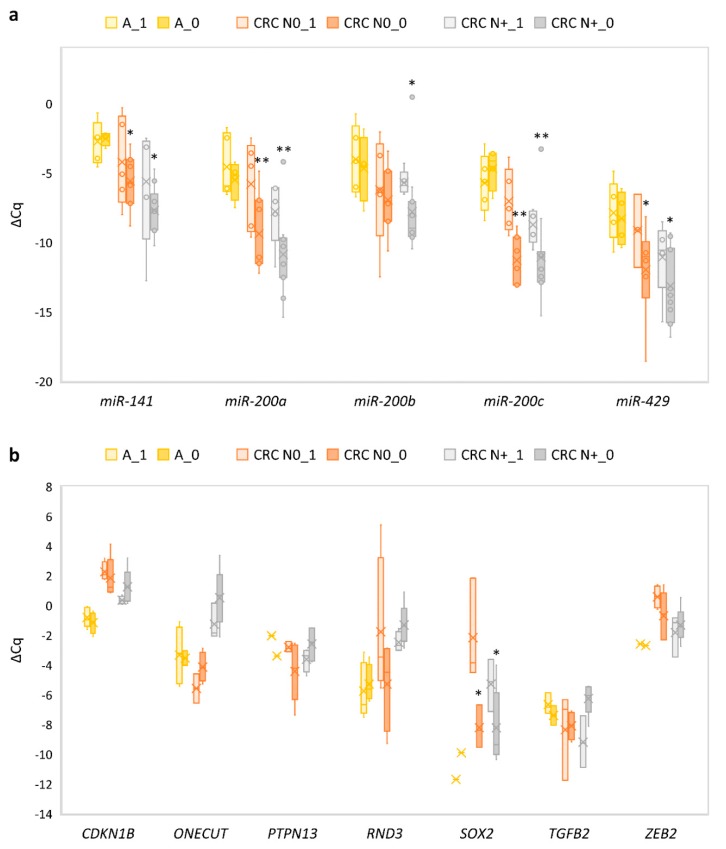
Expression of *miR-200* family and their target genes in adenoma, CRC N0 and CRC N+ based on E-cadherin expression: (**a**) expression of *miR-200* family; (**b**) expression of target genes of *miR-200* family. Legend: CRC, colorectal carcinoma; N0, without nodal metastases; N+, with nodal metastases; 1, preserved expression of E-cadherin (*n* = 6, *n* = 6 and *n* = 8 for adenoma, CRC N0 and CRC N+, respectively); 0, weak or focal loss of E-cadherin (*n* = 4, *n* = 7 and *n* = 9 for adenoma, CRC N0 and CRC N+, respectively); * *p* ≤ 0.05; ** *p* ≤ 0.01.

All miRNAs showed down-regulation and all their target genes showed up-regulation in samples of CRC when comparing those with focal or weak E-cadherin staining with those with preserved staining. A statistically significant change in expression was observed in the case of all miRNAs (*p* = 0.036 for *miR-141*, *p* = 0.003 for *miR-200a*, *p* = 0.014 for *miR-200b*, *p* = 0.003 for *miR-200c*, *p* = 0.034 for *miR-429*) and in the case of SOX2 (*p* = 0.05). Results are summarized in Figure 8a,b.

### 3.11. Correlations between miRNAs and Their Target Genes across All Samples

Numerous correlations were observed between the expression of miRNAs and the expression of their target genes. Surprisingly, correlations between the expression of certain miRNAs and genes that are not yet functionally validated as targets for that particular miRNA, were also observed. All observed correlations are summarized in table (Table 3).

Additionally, the expression of *miR-200b* (r_s_ = −0.499, *p* < 0.001) and *miR-429* (r_s_ = −0.287, *p* = 0.014) were in negative correlation with the severity of the disease (normal mucosa, adenoma, CRC N0 and CRC N+). When samples were divided into only three groups (normal mucosa, adenoma and CRC), a correlation of the severity of the disease was observed with the expression of a higher number of miRNAs and their target genes, namely *miR-200b* (r_s_ = −0.620, *p* < 0.001), *miR-200c* (r_s_ = −0.401, *p* < 0.001), *miR-141* (r_s_ = −0.420, *p* < 0.001), *miR-429* (r_s_ = −0.522, *p* < 0.001), and *CDKN1B* (r_s_ = 0.377, *p* = 0.007), *PTPN13* (r_s_ = −0.426, *p* = 0.006), and *RND3* (r_s_ = 0.467, *p* = 0.016).

## 4. Discussion

EMT has emerged as an important mechanism in cancerogenesis but its role in CRC remains only partially understood. Full EMT is usually observed only in well-controlled experimental conditions, e.g., in cell lines, but it is rarely observed in human tumours [11,16]. We therefore hypothesized that partial EMT, but not full EMT, is induced during CRC cancerogenesis. Our previous studies showed that the use of a single or a few epithelial or mesenchymal markers as a tool for following EMT as a whole is not appropriate [16]. We therefore used several EMT markers (E-cadherin, *miR-200* family and their target genes) in correlation with morphology. Our results support the hypothesis that EMT plays an important role in CRC, both in its development and progression, but only as partial EMT. Our analysis was performed on normal mucosa, adenoma, and carcinoma, including CRC cases with and without nodal metastases, and a comparison between the invasive front and central part of the tumours. 

We first analysed the expression of members of the *miR-200* family, which have been demonstrated as one of the key regulators of EMT in various experimental and human studies [11,16,17]. We found that all five investigated miRNAs were significantly down-regulated in CRC in comparison with adenoma. Both *miR-200a* and *miR-200b* down-regulation have already been described in CRC samples [18,19,20]. However, the results on *miR-141*, *miR-200c* and *miR-429* expression in previous studies are controversial. Whereas, one study described decreased expression of *miR-141* in CRC tissue [21], another study reported up-regulation in CRC compared with adjunct normal mucosa [22]. Similarly, *miR-200c* was observed as up-regulated in some studies [23,24,25] but down-regulated in others [26]. Furthermore, *miR-429* has previously been described as both down-regulated [27] and up-regulated in CRC tissue [28,29]. Our study also showed that all members of the *miR-200* family were up-regulated in adenomas and all, except *miR-200a*, in CRC compared with normal mucosa. In contrast to CRC, there are limited data in the literature on expression of the *miR-200* family in colorectal adenomas and rare published studies have reported that *miR-200a* and *miR-200c* were not significantly changed in adenoma tissue when compared with normal mucosa [30]. 

The finding of a partial EMT induction is further supported by immunohistochemical analysis of E-cadherin, showing focal loss or weak expression in a proportion of adenoma and CRC, and its correlation with down-regulation of all members of the *miR-200* family. Similarly to previously reported studies on E-cadherin expression [31], we found that staining was preserved in all cases of CRC and adenoma, with prevailing membranous immunoreactivity. However, in contrast to our results, a previous study reported that there was no difference between protein expression in tumours and normal mucosa. Another study reported a similar observation as in our study, i.e., E-cadherin expression in all colorectal adenomas and CRC, although it presented with reduced expression in half of them [32]. It has been previously described that *miR-200a* is down-regulated in cells with a reduced expression of E-cadherin protein [18], whereas *miR-200b* decline was not statistically associated with expression of E-cadherin [33]. However, there are limited data about the expression of other members of the *miR-200* family in correlation with E-cadherin protein expression. Our study thus suggests that EMT is induced during CRC cancerogenesis through down-regulation of the *miR-200* family, resulting in a focal loss or weak expression of E-cadherin that might be often observed in partial EMT, in which cells transiently acquire the maximum plasticity and attain hybrid epithelial/mesenchymal phenotype [34].

Though these results strongly suggest induction of EMT in CRC, one criterion for full EMT was not fulfilled, i.e., morphology. When we performed microscopic analysis of CRC and adenoma in comparison with E-cadherin immunohistochemistry, we found that all cases retained an epithelioid morphology, even those with decreased expression of E-cadherin. No spindle cell morphology was found in any case of adenoma or CRC, either in the central parts or at the invasive front. Cell–cell junctions connect epithelial cells, they are polarized, differentiating epithelia from other tissues. In contrast, mesenchymal cells are not polarized and do not possess cell–cell junctions. They are therefore able to migrate through the extracellular matrix, invade and resist apoptosis. During full EMT, epithelial cells undergo extensive changes, they lose polarity, cell–cell junctions (i.e., loss of functional E-cadherin) and reorganize their cytoskeleton, resulting in spindle shaped cells. All these changes lead to a separation of cells and an increase in cell motility at the invasive front [2,5,6,7,8,9,10,11,12]. 

Interestingly, when we compared *miR-200* family expression in the central parts of the tumours and the invasive front, we found their down-regulation at the invasive front in 73% of cases. However, the difference was significant only for *miR-200b*. This finding further supports the concept of ITH even on the level of miRNAs [14]. *miR-200b* was found to be down-regulated in tumour budding cells at the invasive front in 71% of cases [33] and is believed to have a tumour-promoting role in CRC by targeting *RND3* and *CDKN1B* [35]. In addition to *miR-200b*, several published data also described decreased expression of *miR-200c* at the invasive front of the tumour in metastatic CRC cases [36,37,38]. Additionally, *miR-200a/b/c* were also found to be down-regulated at the invasive front of CRC cases with degraded basement membrane [39] but there is limited data in the literature about the expression of *miR-141* and *miR-429* at the invasive front. Down-regulation of the *miR-200* family is believed to be correlated with the loss of the epithelial and gain of the mesenchymal-like phenotype at the invasive front, resulting in increased invasiveness of the CRC tumour cells, thus contributing to migration through the extracellular matrix, colonization of the lymph node and metastatic potential [40]. There is limited data on the expression of coding and/or non-coding genes at the invasive front in comparison with the tumour centre. Previous studies [38,39] and our findings indicate an expression gradient of the *miR-200* family related to ITH. However, recent publications have reported ITH mainly in the context of mutation, copy number variation and methylation status [41,42,43]. ITH includes spatial and temporal ITH, morphological ITH, clonal ITH (derived from genomic instability), and non-clonal ITH (derived from microenvironment interactions). Since ITH is believed to be closely related to cancer progression, resistance to therapy, and recurrence, it is important to consider different types of ITH when investigating mechanisms of cancer progression, prognosis and treatment opportunities [14].

One of the most important aspects of CRC is its metastatic capacities. We therefore analysed the contribution of EMT to the development of nodal metastases. Since invasive properties and metastatic potential are not equivalent functional terms [44], we compared CRC cases without with those witht nodal metastases. We found statistically significant up-regulation of *miR-200b* in CRC with compared with CRC without nodal metastases, implicating miRNAs and possibly EMT in the progression of CRC. This observation and the observed potential invasive role of *miR-200b* in CRC suggest a contribution of *miR-200b* to the invasive and metastatic properties of CRC. Expression of *miR-200b* and *miR-429* were also in correlation with the pTNM stage of CRC, further suggesting a role not only of *miR-200b* but also *miR-429* in CRC progression. It has already been reported that both *miR-200b* and *miR-429* might contribute to the metastatic potential of CRC [20,29,45] and moreover, that up-regulation of *miR-141* contributes to the development of distant metastases in breast cancer [46]. 

We also investigated target genes (*CDKN1B*, *ONECUT2*, *PTPN13*, *RND3*, *SOX2*, *TGFB2*, *WAVE3*, *ZEB1* and *ZEB2*) of the *miR-200* family. Interestingly, only *CDKN1B*, *ONECUT2* and *ZEB2* were differentially expressed in CRC without nodal metastases compared with CRC with nodal metastases. *ZEB2* and *CDKN1B* are targets of *miR-200b* and *ONECUT2* is a target of *miR-429*. Our observation in relation to *CDKN1B* is in accordance with a previously reported study showing that reduced expression of *CDKN1B* is correlated with a poor prognosis for patients with CRC [47]. Using experimental models, it has been shown that *miR-429* reverses TGF-β-induced EMT by interfering with ONECUT2 in CRC cells [26]. However, to the best of our knowledge, this is the first report of ONECUT2 involvement in the human metastatic potential of CRC. In contrast, it has already been reported that ZEB2, which is one of the first identified *miR-200* family targets, promotes tumour metastatic potential and correlates with a poor prognosis for human CRC [48,49,50]. We observed an inverse expression of *miR-200b* and *ZEB2* in CRC with nodal metastases compared with CRC without nodal metastases, further supporting the postulated *ZEB/miR-200* interaction in CRC cancerogenesis [50].

In addition to metastatic potential, all target genes were differentially expressed in CRC compared with normal mucosa. Interestingly, this finding is opposite to that observed in adenoma compared with normal mucosa. The majority of target genes (*CDKN1B*, *PTPN13*, *RND3*, *SOX2* and *ZEB2*) were down-regulated in adenoma compared to normal mucosa. Moreover, *PTPN13*, *SOX2* and *ZEB2* showed detectable expression only in two out of 10 adenoma cases, whereas *ONECUT2* and *TGFB2* were up-regulated. There is limited data on the expression of target genes of the *miR-200* family in colorectal adenoma and, to the best of our knowledge, only *CDKN1B* and *SOX2* have so far been investigated, excluding studies on dysplastic lesions in inflammatory bowel diseases. Immunohistochemistry for *SOX2* has shown that it is expressed in a minority of adenoma cases and all of them were with high-grade dysplasia [51,52]. Similarly, both of our cases that showed expression of *SOX2* (also showing expression of *PTPN13* and *ZEB2*) were adenomas with high-grade dysplasia. Our results thus suggest that an inverse expression of the *miR-200* family and *SOX2* might contribute to the differentiation/proliferation of cells also during CRC cancerogenesis, as already described in neurons [53]. *CDKN1B* was expressed in all cases of adenoma, however, limited published data have reported that *CDKN1B* is not expressed in approx. 20% of adenomas and carcinomas [54] and that its expression does not significantly change during the adenoma–carcinoma sequence/progression of CRC [55]. The only gene that was in correlation to all investigated miRNAs was *RND3*, also known as *RHOE*. *RND3* plays a critical role in arresting cell cycle distribution, inhibiting cell growth and inducing apoptosis and differentiation, and is implicated in processes such as proliferation and migration through cytoskeletal rearrangement. Although it appears that this protein is differently altered according to the tumour context, it has been demonstrated that aberrant *RND3* expression may be the leading cause of tumour metastasis and chemotherapy resistance with a pro-tumourigenic role [56,57]. Accordingly, in tumour and adjacent normal tissues from 202 patients with CRC, including 80 nodal metastases, Rnd3 expression using immunohistochemistry was analysed. Its expression was significantly correlated with depth of invasion, lymph node metastasis and distant metastasis. Most importantly, disease-free and overall survivals were significantly poorer for patients with Rnd3-positive tumours than for those with Rnd3-negative [58]. 

We were not able to detect expression of certain EMT markers. First, we were not able to detect expression of *miR-205* in the tissue samples of normal colon mucosa, adenoma or CRC. Although there are some published studies on *miR-205* expression in experimental models of CRC, only a few of them have described expression in tissue specimens of patients with CRC [59,60,61]. In all of them, *miR-205* was down-regulated, detected on fresh frozen tissues. Members of the *miR-200* family have been observed not only as regulators of EMT but also of EMT-transcriptional factors (EMT-TF), e.g., *ZEB1* and *ZEB2*. However, *ZEB1* was below the detection limit in our samples, whereas *ZEB2* was expressed. In the case of EMT-TFs, there is a possible difference in spatiotemporal expression, depending on the tissue context and tumour type [11]. Members of the same EMT-TF family can even have antagonistic functions. Furthermore, it has been demonstrated that activation of any single EMT-TF is sufficient to induce partial/incomplete EMT and an absence of specific EMT-TF cannot be considered to be proof of the absence of EMT [44]. We were also not able to detect a target gene of *miR-200b*, *WAVE3*, involved in actin cytoskeleton remodelling, participating in the control of cell shape [13].

One of the limitations of our study is a different comparison between adenomas and CRC, and normal mucosa. In excised adenomas (because of endoscopic removal), normal mucosa is often not present, or it is present in very small amounts. In contrast, in patients with CRC, the colon was resected and all resected specimens contained normal mucosa. Each CRC sample was therefore compared to its corresponding normal mucosa as paired tissue samples, using the Wilcoxon Rank test. In contrast, adenomas were compared with normal mucosa samples of CRC resected samples as independent groups of samples, using the Mann–Whitney test. The difference in genetic background, which should be eliminated when comparing paired tissue samples, could lead to overestimation of changes in expression in adenomas in comparison to normal mucosa. Another limitation is related to “normal” samples, which very often present a significant problem in human research. As healthy colon is not resected, truly normal mucosa cannot be obtained. In our study, “normal” samples were taken at least 20 cm away from the tumour and they showed no microscopic abnormalities. However, genetic and protein aberrations may also be present in morphologically normal mucosa [62], although it seems highly unlikely that EMT is activated in such samples. We therefore believe that, despite certain limitations, these samples may be used as corresponding control samples to overcome differences in the genetic background. Moreover, in addition to tumours, various inflammatory diseases, infarction etc. are also an indication for colon surgery. However, when studying EMT, these resection specimens are not suitable for “normal” control, since EMT can also be activated in these diseases.

## 5. Conclusions

Comparing the expression of the *miR-200* family and their target genes in adenoma and CRC with and without nodal metastases showed three patterns. The first pattern was observed in adenoma in comparison with normal mucosa. The second, and opposite to the first, was observed in CRC compared with adenoma and the third pattern was observed in cases of CRC with nodal metastases. Interestingly, all investigated miRNAs were down-regulated in cases with a reduced E-cadherin expression and were mainly down-regulated at the invasive front in comparison with central parts of the tumour. Our results strongly support the postulated hypothesis of partial EMT and ITH during CRC cancerogenesis.

## Figures and Tables

**Table 1:** Probes used for miRNAs and mRNAs quantification using quantitative real-time PCR (qPCR).

Probe Name	Probe ID Number	Length of PCR Product (bp ^1^)
*B2M*	Hs 99999907_m1	75
*CDKN1B*	Hs00153277_m1	71
*IPO8*	Hs 00183533_m1	71
*ONECUT2*	Hs00191477_m1	57
*PTPN13*	Hs01106214_m1	65
*RND3*	Hs01003594_m1	91
*SOX2*	Hs04234836_s1	86
*TGFB2*	Hs01555416_m1	67
*WAVE3*	Hs00903488_m1	57
*ZEB1*	Hs03680599_m1	63
*ZEB2*	Hs01095318_m1	58
*RNU6B*	ID 001093	Nd ^2^
*miR-141*	ID 000463	nd
*miR-200a*	ID 000502	nd
*miR-200b*	ID 002251	nd
*miR-200c*	ID 002300	nd
*miR-205*	ID 000509	nd
*miR-429*	ID 001024	nd
*miR-1274b*	ID 002884	nd

^1^ bp, base pair; ^2^ nd, not defined.

**Table 2:** Patients’ characteristics and results of immunohistochemistry for E-cadherin.

Group	Age (Mean ± SD)	Gender (Male:Female)	pTNM ^1^	No. of Cases with Weak or Focal Loss of Staining of E-Cadherin
**Adenoma (*n* = 10)**	61.00 ± 10.99	10:0	-	4 40.0%
**CRC N0 (*n* = 13)**	74.62 ± 11.09	4:9	pT1N0 (*n* = 1) pT2N0 (*n* = 2) pT3N0 (*n* = 8) pT4N0 (*n* = 2)	7 53.9%
**CRC N+ (*n* = 17)**	70.88 ± 13.87	8:9	pT3N1 (*n* = 6) pT4N1 (*n* = 4) pT4N2 (*n* = 7)	9 52.9%

**^1^** pathologic Tumor Node Metastasis classification [15].

**Table 3:** Statistically significant correlations between expression of *miR-200* family and their target genes and association with E-cadherin staining.

Correlations	*CDKN1B*	*ONECUT2*	*PTPN13*	*RND3*	*SOX2*	*TGFB2*	*ZEB2*	E-Cadherin
***miR-200a***	−0.360	−0.395	/	−0.325	/	/	/	0.474
***miR-200b***	/	/	/	−0.384	/	/	/	0.400
***miR-200c***	−0.503	/	/	−0.337	/	/	/	0.484
***miR-141***	/	/	/	−0.351	/	/	/	0.355
***miR-429***	/	/	/	−0.347	/	/	/	0.363
**E-cadherin**	/	/	/	/	0.521	/	/	**1**

## References

[1] Balch C., Ramapuram J.B., Tiwari A.K. (2017). The epigenomics of embryonic pathway signaling in colorectal cancer. Front. Pharmacol..

[2] Cao H., Xu E., Liu H., Wan L., Lai M. (2015). Epithelial-mesenchymal transition in colorectal cancer metastasis: A system review. Pathol. Res. Pract..

[3] Nazemalhosseini Mojarad E., Kuppen P.J., Aghdaei H.A., Zali M.R. (2013). The CpG island methylator phenotype (CIMP) in colorectal cancer. Gastroenterol. Hepatol. Bed Bench.

[4] Kudryavtseva A.V., Lipatova A.V., Zaretsky A.R., Moskalev A.A., Fedorova M.S., Rasskazova A.S., Shibukhova G.A., Snezhkina A.V., Kaprin A.D., Alekseev B.Y. (2016). Important molecular genetic markers of colorectal cancer. Oncotarget.

[5] Goossens S., Vandamme N., Van Vlierberghe P., Berx G. (2017). EMT transcription factors in cancer development re-evaluated: Beyond EMT and MET. Biochim. Biophys. Acta Rev. Cancer.

[6] Iwatsuki M., Mimori K., Yokobori T., Ishi H., Beppu T., Nakamori S., Baba H., Mori M. (2010). Epithelial-mesenchymal transition in cancer development and its clinical significance. Cancer Sci..

[7] Prieto-Garcia E., Diaz-Garcia C.V., Garcia-Ruiz I., Agullo-Ortuno M.T. (2017). Epithelial-to-mesenchymal transition in tumor progression. Med. Oncol..

[8] Acloque H., Thiery J.P., Nieto M.A. (2008). The physiology and pathology of the EMT. Meeting on the epithelial-mesenchymal transition. EMBO Rep..

[9] Bryant D.M., Mostov K.E. (2008). From cells to organs: Building polarized tissue. Nat. Rev. Mol. Cell Biol..

[10] Gurzu S., Silveanu C., Fetyko A., Butiurca V., Kovacs Z., Jung I. (2016). Systematic review of the old and new concepts in the epithelial-mesenchymal transition of colorectal cancer. World J. Gastroenterol..

[11] Stemmler M.P., Eccles R.L., Brabletz S., Brabletz T. (2019). Non-redundant functions of EMT transcription factors. Nat. Cell Biol..

[12] Findlay V.J., Wang C., Watson D.K., Camp E.R. (2014). Epithelial-to-mesenchymal transition and the cancer stem cell phenotype: Insights from cancer biology with therapeutic implications for colorectal cancer. Cancer Gene Ther..

[13] Humphries B., Yang C. (2015). The microRNA-200 family: Small molecules with novel roles in cancer development, progression and therapy. Oncotarget.

[14] Stanta G., Bonin S. (2018). Overview on clinical relevance of intra-tumor heterogeneity. Front. Med. (Lausanne).

[15] Brierley J.D., Gospodarowicz M.K., Wittekind C. (2017). TNM Classification of Malignant Tumours.

[16] Zidar N., Bostjancic E., Gale N., Kojc N., Poljak M., Glavac D., Cardesa A. (2011). Down-regulation of microRNAs of the miR-200 family and miR-205, and an altered expression of classic and desmosomal cadherins in spindle cell carcinoma of the head and neck--hallmark of epithelial-mesenchymal transition. Hum. Pathol..

[17] Zidar N., Bostjancic E., Jerala M., Kojc N., Drobne D., Stabuc B., Glavac D. (2016). Down-regulation of microRNAs of the miR-200 family and up-regulation of Snail and Slug in inflammatory bowel diseases—Hallmark of epithelial-mesenchymal transition. J. Cell. Mol. Med..

[18] Pichler M., Ress A.L., Winter E., Stiegelbauer V., Karbiener M., Schwarzenbacher D., Scheideler M., Ivan C., Jahn S.W., Kiesslich T. (2014). MiR-200a regulates epithelial to mesenchymal transition-related gene expression and determines prognosis in colorectal cancer patients. Br. J. Cancer.

[19] Liang W.C., Fu W.M., Wong C.W., Wang Y., Wang W.M., Hu G.X., Zhang L., Xiao L.J., Wan D.C., Zhang J.F. (2015). The lncRNA H19 promotes epithelial to mesenchymal transition by functioning as miRNA sponges in colorectal cancer. Oncotarget.

[20] Lv Z., Wei J., You W., Wang R., Shang J., Xiong Y., Yang H., Yang X., Fu Z. (2017). Disruption of the c-Myc/miR-200b-3p/PRDX2 regulatory loop enhances tumor metastasis and chemotherapeutic resistance in colorectal cancer. J. Transl. Med..

[21] Feng L., Ma H., Chang L., Zhou X., Wang N., Zhao L., Zuo J., Wang Y., Han J., Wang G. (2016). Role of microRNA-141 in colorectal cancer with lymph node metastasis. Exp. Ther. Med..

[22] Ding L., Yu L.L., Han N., Zhang B.T. (2017). miR-141 promotes colon cancer cell proliferation by inhibiting MAP2K4. Oncol. Lett..

[23] Wang M., Zhang P., Li Y., Liu G., Zhou B., Zhan L., Zhou Z., Sun X. (2012). The quantitative analysis by stem-loop real-time PCR revealed the microRNA-34a, microRNA-155 and microRNA-200c overexpression in human colorectal cancer. Med. Oncol..

[24] Chen J., Wang W., Zhang Y., Hu T., Chen Y. (2014). The roles of miR-200c in colon cancer and associated molecular mechanisms. Tumour Biol..

[25] Roh M.S., Lee H.W., Jung S.B., Kim K., Lee E.H., Park M.I., Lee J.S., Kim M.S. (2018). Expression of miR-200c and its clinicopathological significance in patients with colorectal cancer. Pathol. Res. Pract..

[26] Lu Y.X., Yuan L., Xue X.L., Zhou M., Liu Y., Zhang C., Li J.P., Zheng L., Hong M., Li X.N. (2014). Regulation of colorectal carcinoma stemness, growth, and metastasis by an miR-200c-Sox2-negative feedback loop mechanism. Clin. Cancer Res..

[27] Sun Y., Shen S., Liu X., Tang H., Wang Z., Yu Z., Li X., Wu M. (2014). MiR-429 inhibits cells growth and invasion and regulates EMT-related marker genes by targeting Onecut2 in colorectal carcinoma. Mol. Cell. Biochem..

[28] Li J., Du L., Yang Y., Wang C., Liu H., Wang L., Zhang X., Li W., Zheng G., Dong Z. (2013). MiR-429 is an independent prognostic factor in colorectal cancer and exerts its anti-apoptotic function by targeting SOX2. Cancer Lett..

[29] Han Y., Zhao Q., Zhou J., Shi R. (2017). miR-429 mediates tumor growth and metastasis in colorectal cancer. Am. J. Cancer Res..

[30] Wang X., Chen L., Jin H., Wang S., Zhang Y., Tang X., Tang G. (2016). Screening miRNAs for early diagnosis of colorectal cancer by small RNA deep sequencing and evaluation in a Chinese patient population. Onco Targets Ther..

[31] Bezdekova M., Brychtova S., Sedlakova E., Langova K., Brychta T., Belej K. (2012). Analysis of Snail-1, E-cadherin and claudin-1 expression in colorectal adenomas and carcinomas. Int. J. Mol. Sci..

[32] Kroepil F., Fluegen G., Totikov Z., Baldus S.E., Vay C., Schauer M., Topp S.A., Esch J.S., Knoefel W.T., Stoecklein N.H. (2012). Down-regulation of CDH1 is associated with expression of SNAI1 in colorectal adenomas. PLoS ONE.

[33] Knudsen K.N., Lindebjerg J., Nielsen B.S., Hansen T.F., Sorensen F.B. (2017). MicroRNA-200b is downregulated in colon cancer budding cells. PLoS ONE.

[34] Jolly M.K., Mani S.A., Levine H. (2018). Hybrid epithelial/mesenchymal phenotype(s): The ‘fittest’ for metastasis?. Biochim. Biophys. Acta Rev. Cancer.

[35] Fu Y., Liu X., Zhou N., Du L., Sun Y., Zhang X., Ge Y. (2014). MicroRNA-200b stimulates tumour growth in TGFBR2-null colorectal cancers by negatively regulating p27/kip1. J. Cell. Physiol..

[36] Hur K., Toiyama Y., Takahashi M., Balaguer F., Nagasaka T., Koike J., Hemmi H., Koi M., Boland C.R., Goel A. (2013). MicroRNA-200c modulates epithelial-to-mesenchymal transition (EMT) in human colorectal cancer metastasis. Gut.

[37] Muto Y., Suzuki K., Kato T., Tsujinaka S., Ichida K., Takayama Y., Fukui T., Kakizawa N., Watanabe F., Saito M. (2016). Heterogeneous expression of zinc-finger E-box-binding homeobox 1 plays a pivotal role in metastasis via regulation of miR-200c in epithelial-mesenchymal transition. Int. J. Oncol..

[38] Jepsen R.K., Novotny G.W., Klarskov L.L., Christensen I.J., Hogdall E., Riis L.B. (2016). Investigating intra-tumor heterogeneity and expression gradients of miR-21, miR-92a and miR-200c and their potential of predicting lymph node metastases in early colorectal cancer. Exp. Mol. Pathol..

[39] Paterson E.L., Kazenwadel J., Bert A.G., Khew-Goodall Y., Ruszkiewicz A., Goodall G.J. (2013). Down-regulation of the miRNA-200 family at the invasive front of colorectal cancers with degraded basement membrane indicates EMT is involved in cancer progression. Neoplasia.

[40] Davalos V., Moutinho C., Villanueva A., Boque R., Silva P., Carneiro F., Esteller M. (2012). Dynamic epigenetic regulation of the microRNA-200 family mediates epithelial and mesenchymal transitions in human tumorigenesis. Oncogene.

[41] Naxerova K., Reiter J.G., Brachtel E., Lennerz J.K., van de Wetering M., Rowan A., Cai T., Clevers H., Swanton C., Nowak M.A. (2017). Origins of lymphatic and distant metastases in human colorectal cancer. Science.

[42] Saito T., Niida A., Uchi R., Hirata H., Komatsu H., Sakimura S., Hayashi S., Nambara S., Kuroda Y., Ito S. (2018). A temporal shift of the evolutionary principle shaping intratumor heterogeneity in colorectal cancer. Nat. Commun..

[43] Uchi R., Takahashi Y., Niida A., Shimamura T., Hirata H., Sugimachi K., Sawada G., Iwaya T., Kurashige J., Shinden Y. (2016). Integrated multiregional analysis proposing a new model of colorectal cancer evolution. PLoS Genet..

[44] Brabletz T., Kalluri R., Nieto M.A., Weinberg R.A. (2018). EMT in cancer. Nat. Rev. Cancer.

[45] Sun Y., Shen S., Tang H., Xiang J., Peng Y., Tang A., Li N., Zhou W., Wang Z., Zhang D. (2014). miR-429 identified by dynamic transcriptome analysis is a new candidate biomarker for colorectal cancer prognosis. OMICS.

[46] Debeb B.G., Lacerda L., Anfossi S., Diagaradjane P., Chu K., Bambhroliya A., Huo L., Wei C., Larson R.A., Wolfe A.R. (2016). miR-141-mediated regulation of brain metastasis from breast cancer. J. Natl. Cancer Inst..

[47] Li J.Q., Miki H., Wu F., Saoo K., Nishioka M., Ohmori M., Imaida K. (2002). Cyclin A correlates with carcinogenesis and metastasis, and p27(kip1) correlates with lymphatic invasion, in colorectal neoplasms. Hum. Pathol..

[48] Sreekumar R., Harris S., Moutasim K., DeMateos R., Patel A., Emo K., White S., Yagci T., Tulchinsky E., Thomas G. (2018). Assessment of nuclear ZEB2 as a biomarker for colorectal cancer outcome and TNM risk stratification. JAMA Netw. Open.

[49] Li M.Z., Wang J.J., Yang S.B., Li W.F., Xiao L.B., He Y.L., Song X.M. (2017). ZEB2 promotes tumor metastasis and correlates with poor prognosis of human colorectal cancer. Am. J. Transl. Res..

[50] Brabletz S., Brabletz T. (2010). The ZEB/miR-200 feedback loop--a motor of cellular plasticity in development and cancer?. EMBO Rep..

[51] Talebi A., Kianersi K., Beiraghdar M. (2015). Comparison of gene expression of SOX2 and OCT4 in normal tissue, polyps, and colon adenocarcinoma using immunohistochemical staining. Adv. Biomed. Res..

[52] Miller T.J., McCoy M.J., Hemmings C., Iacopetta B., Platell C.F. (2018). Expression of PD-L1 and SOX2 during rectal tumourigenesis: Potential mechanisms for immune escape and tumour cell invasion. Oncol. Lett..

[53] Pandey A., Singh P., Jauhari A., Singh T., Khan F., Pant A.B., Parmar D., Yadav S. (2015). Critical role of the miR-200 family in regulating differentiation and proliferation of neurons. J. Neurochem..

[54] Arber N., Hibshoosh H., Yasui W., Neugut A.I., Hibshoosh A., Yao Y., Sgambato A., Yamamoto H., Shapira I., Rosenman D. (1999). Abnormalities in the expression of cell cycle-related proteins in tumors of the small bowel. Cancer Epidemiol. Biomark. Prev..

[55] Ohuchi M., Sakamoto Y., Tokunaga R., Kiyozumi Y., Nakamura K., Izumi D., Kosumi K., Harada K., Kurashige J., Iwatsuki M. (2018). Increased EZH2 expression during the adenoma-carcinoma sequence in colorectal cancer. Oncol. Lett..

[56] Jie W., Andrade K.C., Lin X., Yang X., Yue X., Chang J. (2015). Pathophysiological functions of Rnd3/RhoE. Compr. Physiol..

[57] Paysan L., Piquet L., Saltel F., Moreau V. (2016). Rnd3 in Cancer: A Review of the evidence for tumor promoter or suppressor. Mol. Cancer Res..

[58] Zhou J., Yang J., Li K., Mo P., Feng B., Wang X., Nie Y., Fan D. (2013). RhoE is associated with relapse and prognosis of patients with colorectal cancer. Ann. Surg. Oncol..

[59] Orang A.V., Safaralizadeh R., Hosseinpour Feizi M.A., Somi M.H. (2014). Diagnostic and prognostic value of miR-205 in colorectal cancer. Asian Pac. J. Cancer Prev..

[60] Li P., Xue W.J., Feng Y., Mao Q.S. (2015). MicroRNA-205 functions as a tumor suppressor in colorectal cancer by targeting cAMP responsive element binding protein 1 (CREB1). Am. J. Transl. Res..

[61] Boulagnon-Rombi C., Schneider C., Leandri C., Jeanne A., Grybek V., Bressenot A.M., Barbe C., Marquet B., Nasri S., Coquelet C. (2018). LRP1 expression in colon cancer predicts clinical outcome. Oncotarget.

[62] Polley A.C., Mulholland F., Pin C., Williams E.A., Bradburn D.M., Mills S.J., Mathers J.C., Johnson I.T. (2006). Proteomic analysis reveals field-wide changes in protein expression in the morphologically normal mucosa of patients with colorectal neoplasia. Cancer Res..

